# Urinary tract infection among obstetric fistula patients at Gondar University Hospital, Northwest Ethiopia

**DOI:** 10.1186/1472-6874-14-12

**Published:** 2014-01-17

**Authors:** Yitayih Wondimeneh, Dagnachew Muluye, Abebe Alemu, Asmamaw Atinafu, Gashaw Yitayew, Teklay Gebrecherkos, Agersew Alemu, Demekech Damtie, Getachew Ferede

**Affiliations:** 1 School of Biomedical and Laboratory Sciences, College of Medicine and Health Sciences, University of Gondar, P.O. Box 196, Gondar, Ethiopia; 2 Bahir Dar Regional Health Research Laboratory Center, P.O. Box 641, Bahir Dar, Ethiopia

**Keywords:** UTI, Obstetric Fistula, Gondar University hospital

## Abstract

**Background:**

Many women die from complications related to pregnancy and childbirth. In developing countries particularly in sub-Saharan Africa and Asia, where access to emergency obstetrical care is often limited, obstetric fistula usually occurs as a result of prolonged obstructed labour. Obstetric fistula patients have many social and health related problems like urinary tract infections (UTIs). Despite this reality there was limited data on prevalence UTIs on those patients in Ethiopia. Therefore, the aim of this study was to determine the prevalence, drug susceptibility pattern and associated risk factors of UTI among obstetric fistula patients at Gondar University Hospital, Northwest Ethiopia.

**Methods:**

A cross sectional study was conducted from January to May, 2013 at Gondar University Hospital. From each post repair obstetric fistula patients, socio-demographic and UTIs associated risk factors were collected by using a structured questionnaire. After the removal of their catheters, the mid-stream urine was collected and cultured on CLED. After overnight incubation, significant bacteriuria was sub-cultured on Blood Agar Plate (BAP) and MacConkey (MAC). The bacterial species were identified by series of biochemical tests. Antibiotic susceptibility test was done by disc diffusion method. Data was entered and analyzed by using SPSS version 20.

**Results:**

A total of 53 post repair obstetric fistula patients were included for the determination of bacterial isolate and 28 (52.8%) of them had significant bacteriuria. Majority of the bacterial isolates, 26 (92.9%), were gram negative bacteria and the predominant ones were *Citrobacter* 13 (24.5%) and *E. coli* 6 (11.3%). *Enterobacter, E.coli* and *Proteus mirabilis* were 100% resistant to tetracycline. *Enterobacter, Proteus mirabilis, Klebsella pneumonia, Klebsella ozenae* and *Staphylococcus aureus* were also 100% resistant to ceftriaxone.

**Conclusion:**

The prevalence of bacterial isolates in obstetric fistula patients was high and majority of the isolates were gram negative bacteria. Even thought the predominant bacterial isolates were *Citrobacter* and *E. coli,* all of the bacterial isolates had multiple antibiotic resistance patterns which alert health profession to look better treatment for these patients.

## Background

Globally, more than half a million young women die from complications of pregnancy and childbirth in each year and almost all these deaths occur in developing countries [1]. In resource-poor settings, where access to emergency obstetrical care is often limited, obstetric fistula (OF) usually occurs as a result of prolonged obstructed labour especially in adolescent girls. In parts of sub-Saharan Africa and Asia, it is estimated that more than 2 million young women live with untreated obstetric fistula and between 50, 000 and 100, 000 new women are affected each year [2]. However, many women are still unaware of the availability of treatment and 80% of them never seek treatment due to lack of knowledge [3].

Patients with obstetric fistula can have frequent bladder infections, incontinence of urine and stool. Many of these patients might live with these conditions for several years. This may further predispose them to health related problems like urinary tract infections (UTI) [3,4]. Urinary tract infection is one of the most common infectious diseases and it is a serious ailment in human due to the frequency, recurrence and difficulty in eradication [5,6].

After surgical procedures, catheterization is common in most obstetric fistula patients to empty the bladder [7]. This post repaired catheterization may provide an opportunity for the adequate tensile strength and wound healing [8]. However, the post-repair urethral catheterization may be associated with increased risk of healthcare-associated infection in obstetric fistula patients [9]. In addition, these obstetric fistula patients may also develop several complications such as urethral bleeding, urethritis, urethral stricture, bladder stones and urinary tract infections [10,11]. In general, obstetric fistula treatment needs prolonged hospitalization and more intensive nursing cares like prolonged bladder catheterization which may contribute for the development of urinary tract infections in women who have OF [12]. In Ethiopia, it is estimated that 9 000 women annually develop a fistula [13]. However, little is known about the impact of UTI on obstetric fistula patients in the country. Data on the current distribution and antimicrobial drug susceptibility patterns among urinary bacterial isolates from such patients in the study area is also scarce. Therefore, the aim of this study was to determine the prevalence, antimicrobial susceptibility and UTI associated risk factors among obstetric fistula patients at Gondar University Hospital, Northwest Ethiopia.

## Methods

### Study design, area and period

A cross sectional study was conducted from January to May 2013 at Gondar University Hospital, Northwest Ethiopia. Gondar University Hospital is located 738 kilometers far from the capital city, Addis Ababa. This University Hospital is not only acts as a teaching hospital but also used as a referral hospital for the surrounding regions. The hospital has specializations in internal medicine, pediatrics, gynecology, surgery, and other health related specializations and it serves for greater than five million people in inpatient and outpatient departments. It has also well organized fistula clinic that can serve not only for the large community of Amhara but also for the nearby regions.

### Study participants and data collection

The study participants were all post repair fistula patients who have attended Gondar University Hospital fistula clinic during the study period. Study participants were interviewed about the presence and duration of their clinical manifestation of UTI. Socio-demographic characteristics and other UTI related risk factors of study participants were collected using structured questionnaires and the physical examination was done by a physician working at fistula clinic.

### Collection, handling and transport of urine specimen

After removing their catheters, each post repair fistula patient was informed by the attending nurses how to collect a ‘clean-catch’ mid stream urine specimen in the sterile bottle container. Accordingly, about 10 to 20 ml urine specimen was collected in a sterile screw-capped, wide-necked test tube. The specimen was immediately delivered to the hospital laboratory bacteriology unit for laboratory analysis.

### Culture and identification

After laboratory arrival, the specimen was cultured on cysteine lactose electrolyte deficient agar (CLED) by using a calibrated loop that can deliver 0.002 ml of urine. The streaked culture CLED tube was overnight incubated at 37°C. On the next day, a single colony was picked from culture plates with significant bacteriuria (10^5^ colony forming units per ml of urine) and suspended in nutrient broth, and sub cultured onto blood agar and MacConkey agar and finally incubated at 37°C for further purification. Pure isolates of bacterial pathogen was preliminary characterized by colony morphology, gram-stain, and catalase test. A standard biochemical procedure was used for full identification of gram- positive and gram negative bacteria.

### Antimicrobial susceptibility testing

Antimicrobial susceptibility tests were done on Mueller-Hinton agar (Oxoid, England) using disk diffusion method [14]. The antimicrobial agents tested were Ampicillin (10 μg), Amoxycillin (10 μg), Ceftriaxone (30 μg), Chloramphenicol (30 μg), Ciprofloxacin (5 μg), Gentamycin (10 μg), Norfluxaciline (10 μg), Cotrimoxazole (25 μg), Tetracycline (30 μg), Nitrofurantion (300 μg). The drug susceptibility pattern was interpreted according to Clinical and Laboratory Standards Institute (CLSI, 2006) (formerly known as National Committee for Clinical Laboratory Standards/NCCLS) [15]. Reference strains of *E. coli* ATCC 25922 and *S. aureus* ATCC 25923 were used for quality control for antimicrobial susceptibility tests [15].

### Data analysis procedure

Data were cleaned manually and entered and analyzed by using SPSS version 20 software. Chi-square test with 95% CI was employed to compare the proportion of bacterial isolates with patients’ demographic information and comparison of antimicrobial resistances. P-value ≤ 0.05 was considered statistically significant.

### Ethical considerations

This research project was done after obtaining Institutional Ethical Clearance from the Ethical Committee of University of Gondar. Informed consent was obtained from each study participants before collecting socio demographic data and urine specimens for laboratory analysis.

## Results

During the study period, a total of 53 obstetric fistula patients were attended the clinic and all of them were participated in the study. The mean age of the study participants was 28.6 ± 10.8 years with minimum and maximum age of 13 and 65 years respectively. Majority of the study participants 19 (35.8%) were in the age group of 21-30 years and 44 (83%) were from rural settings. A high proportion 37 (69.8%) of the study participants were married, 42 (79.2%) were illiterate and 33 (62.3%) were house wives (Table 1).

**Table 1:** Significant bacterial isolate in relation to socio-demographic characteristics and associated factors of obstetric fistula patients at Gondar University Hospital, Northwest Ethiopia, 2013

**Variables**	**Significant bacterial isolated N (%)**			**P-value**
	**Yes**	**No**	**Total**	
**Age in years**				
10–20	7 (46.7)	8 (53.3)	15 (28.3)	0.886
21–30	10 (52.6)	9 (47.4)	19 (35.9)	
31–40	8 (61.5)	5 (38.5)	13 (24.5)	
>40	3 (50.0)	3 (50.0)	6 (11.3)	
**Residence**				
Urban	5 (55.6)	4 (44.4)	9 (17.0)	0.857
Rural	25 (56.8)	19 (43.2)	44 (83.0)	
**Marital status**				
Single	0 (0.0)	4 (100.0)	4 (7.5)	0.032
Married	19 (51.4)	18 (48.6)	37 (69.8)	
Divorced	9 (75.0)	3 (25.0)	12 (22.6)	
**Education**				
Illiterate	23 (54.8)	19 (45.2)	42 (79.2)	0.582
Primary school	5 (45.5)	6 (54.5)	11 (20.8)	
**Occupation**				
Merchant	2 (66.7)	1 (33.3)	3 (5.7)	0.169
Farmer	7 (77.8)	2 (22.2)	9 (17.0)	
Student	1 (33.3)	2 (66.7)	3 (5.70	
House wife	15 (45.5)	18 (54.5)	33 (62.3)	
Daily laborer	2 (40.0)	3 (60.0)	5 (9.4)	
**Prev. hist. of catheterization**				
Yes	21 (56.8)	16 (43.2)	37 (69.8)	0.384
No	7 (43.8)	9 (56.2)	16 (30.2)	

**Key**: Prev. hist. of catheterization - Previous history of catheterization before seeking obstetric fistula treatment center.

The overall prevalence of bacterial isolate was 28 (52.8%) and 10 (62.5%) of the isolates were from symptomatic cases. Majority of the bacterial isolates 8 (61.5%) were in the age groups of 31-40 years (P = 0.886). A high prevalence of significant bacteriuria was also isolated from divorced 9 (75.0%) (P = 0.032), illiterate 23 (54.8%) and farmer 7 (77.8%) study participants. Majority of the study participants 33 (62.3%) had previous history of catheterization (Table 1). Eight (15.1%) and 20 (37.7%) of the obstetric fistula patients had history of supra-pubic pain and irritative voiding symptoms, respectively. They had also history of dysuria (3.8%) and urethral strictures (1.9%) (Table 2).

**Table 2:** Clinical presentation of obstetric fistula patients at Gondar University Hospital, Northwest Ethiopia, 2013

**Clinical presentation and risk factors**	**Frequency N (%)**	
	**Yes**	**No**
History of suprapubic pain	8 (15.1)	45 (84.9)
History of irritative voiding symptoms	20 (37.7)	33 (62.3)
History of intestinal motility disorders	7 (13.2)	46 (86.8)
History of dysuria	2 (3.8)	51 (96.2)
History of urethral strictures	1 (1.9)	52 (98.1)

In this study, majority 26 (92.9%) of the bacterial isolates were gram negative bacteria and the predominant isolates were *Citrobacter* 13 (24.5%) and *E. coli* 6 (11.3%) (Table 3). *E. coli, Enterobacter* and *Proteus mirabilis* were 100% resistant to TTC. Furthermore, *Enterobacter, Proteus mirabilis, Klebsella pneumonia, Klebsella ozenae* and *Staphylococcus aureus* were also 100% resistant to CRO (Table 4).

**Table 3:** Bacterial isolates from obstetric fistula patients at Gondar University Hospital, Northwest Ethiopia, 2013

**Bacterial isolates**	**Frequency**	**Percent**
*Staphylococcus aureus*	2	3.8
*Escherchia coli*	6	11.3
*Citrobacter*	13	24.5
*Enterobacter*	3	5.7
*Proteus mirabilis*	1	1.9
*Klebsella pneumonia*	1	1.9
*Klebsella ozenae*	2	3.8

**Table 4:** Antimicrobial susceptibility pattern of bacterial isolates from obstetric fistula patients at Gondar University Hospital, Northwest Ethiopia, 2013

**Bacterial isolates**		**Antimicrobial agents**								
		**AMP**	**AMX**	**CRO**	**CAF**	**CIP**	**CN**	**NOR**	**SXT**	**TTC**
*E. coli*	S	2 (33.3)	3 (50.0)	3 (50.0)	5 (83.3)	3 (50.0)	2 (33.3)	2 (33.3)	2 (33.3)	0 (0.0)
	R	4 (66.7)	3 (50.0)	3 (50.0)	1 (16.7)	3 (50.0)	4 (66.7)	4 (66.7)	4 (66.7)	6 (100.0)
*Citrobacter*	S	4 (30.8)	4 (30.8)	7 (53.8)	3 (23.1)	6 (46.2)	5 (38.5)	6 (46.2)	5 (38.5)	5 (38.5)
	R	9 (69.2)	9 (69.2)	6 (46.2)	10 (76.9)	7 (53.8)	8 (61.5)	7 (53.8)	8 (61.5)	8 (61.5)
*Enterobacter*	S	2 (66.7)	1 (33.3)	0 (0.0)	0 (0.0)	2 (66.7)	19 (33.3)	2 (66.7)	2 (66.7)	0 (0.0)
	R	1 (33.3)	2 (66.7)	3 (100.0)	3 (100.0)	1 (33.3)	2 (66.7)	1 (33.3)	1 (33.3)	3 (100.0)
*Proteus mirabilis*	S	0 (0.0)	0 (0.0)	0 (0.0)	1 (100.0)	0 (0.0)	0 (0.0)	1 (100.0)	0 (0.0)	0 (0.0)
	R	1 (100.0)	1 (100.0)	1 (100.0)	0 (0.0)	1 (100.0)	1 (100.0)	0 (0.0)	1 (100.0)	1 (100.0)
*Klebsella pneumonia*	S	1 (100.0)	0 (0.0)	0 (0.0)	1 (100.0)	0 (0.0)	1 (100.0)	0 (0.0)	1 (100.0)	1 (100.0)
	R	0 (0.0)	1 (100.0)	1 (100.0)	0 (0.0)	1 (100.0)	0 (0.0)	1 (100.0)	0 (0.0)	0 (0.00
*Klebsella ozenae*	S	1 (50.0)	0 (0.0)	0 (0.0)	1 (50.0)	1 (50.0)	1 (50.0)	2 (100.0)	0 (0.0)	1 (50.0)
	R	1 (50.0)	2 (100.0)	2 (100.0)	1 (50.0)	1 (50.0)	1 (50.0)	0 (0.0)	2 (100.0)	1 (50.0)
*Staphylococcus aureus*	S	0 (0.0)	0 (0.0)	0 (0.0)	1 (50.0)	0 (0.0)	1 (50.0)	0 (0.0)	0 (0.0)	2 (100.0)
	R	2 (100.0)	2 (100.0)	2 (100.0)	1 (50.0)	2 (100.0)	1 (50.0)	2 (100.0)	2 (100.0)	0 (0.0)

**Keys:***AMP*, ampicillin, *AMX*, amoxacillin, *CRO*, ceftriaxone, *CAF*, chloramphenicol, *CIP*, ciprofloxacin, *CN*, gentamycin, *NOR*, norfluxaciline, *SXT*, co-trimoxazole, *TTC*, tetracycline.

## Discussion

Though UTI is common in both men and women, there is a large difference in UTI prevalence between them due to variety of factors [16]. Obstetric fistula is one of the risk factors for the development of UTI in women. But there is a lack of concrete evidences that show the magnitude of UTI and antimicrobial sensitivity pattern in obstetric fistula patients throughout the world and it is difficult to compare all the current findings with previous reports. In the present study, the overall prevalence of UTI was 28 (52.8%) which is almost similar with other report in Nigeria (76.1%) [17]. But this finding was higher than other studies in Kenya 8.0% [18] and Nigeria 11 (9.2%) [19]. The prevalence of UTI in the present study was also higher than another reports from pregnant women in the study area [20], Addis Ababa Ethiopia [21] and Tanzania [22]. This difference in similar study participants may be due to the difference in the risk factors like history of catheterization and immune system of the study participants. As indicated in Table 1, majority of the study participants had previous history of catheterization before seeking obstetric fistula treatment and this factor might increase the development of UTIs.

In the present study, the prevalence of symptomatic and asymptomatic bacteriuria were 10 (62.5%) and 18 (48.6%), respectively. Similar findings were also reported in previous studies in Ethiopia [20] and Tanzania [22] from pregnant women. In this study, a statically significant bacteriuria was found among divorced study participants. This might be associated with the study participant psychological readiness to keep their personal hygiene. In addition to developing obstetric fistula, divorcing may upset them to be hopeless and they may not be ready to accept it and developed mental trauma and they may not bother about their personal hygiene. Furthermore, they may not know how to keep their personal hygiene due to lack of knowledge. But there is no previous supportive data regarding to these issues and it needs further studies.

In the present study, the predominant bacterial isolates were gram negative which was similar to other findings in the study area [20] and Addis Ababa, Ethiopia [21] from pregnant women. In this study, the most common bacterial isolates were *Citrobacter* and *E. coli*. A similar high proportion of *E. coli* was also reported in Sudan [23] and Yemen [24] studies. Though *E. coli* is considered the most uropathogenic due to its virulence factors for colonization and invasion of the urinary epithelium [25], *Citrobacter* can be also the most important uropathogens in the study area but it needs further study on its epidemiology and risk factors.

The majority of the bacterial isolates were from rural settings and illiterate study participants since these study groups may not have awareness how to keep their personal hygiene. Similarly, these study participants may lived for several years with obstetric fistula before treatment and this might increase the development of UTI in these study groups. However, there is no previous data regarding to this issue and it needs further study on different settings with a different study design like longitudinal comparative study.

Prescribing and giving antibiotics without testing antibiotic resistance pattern in developing countries including Ethiopia is a common problem for the development of drug resistance. In the present study, majority of the bacterial isolates were highly resistant to tetracycline and ceftriaxone. A similar finding was also reported in other studies [26,27]. This high antibiotic resistance might be associated with previous exposure of the bacterial isolates to these antibiotics. In addition, in rural settings including the study area, use of antibiotics without proper prescription with health professionals is also a common practice. All these factors may be used as a risk factor for the development of antibiotic resistance especially for the commonly used ones. Furthermore, all of the bacterial isolates in this study were multidrug resistant.

## Conclusion

In conclusion, the prevalence of bacterial isolates in obstetric fistula patients was high and majority of the isolates were gram negative bacteria. The predominant bacterial isolates were *Citrobacter* and *E. coli*. All of the bacterial isolates had multiple antibiotic resistance patterns. Therefore antibiotic susceptibility test is mandatory before prescribing any antibiotic drugs.

### Limitation

Due to the small sample size, it may be difficult to conclude the magnitude of UTI problems on obstetric fistula patients. In addition, the clinical presentation and risk factors such as prolonged catheterization, presence of urinary retention, urethral instrumentation, poor immune system and concurrent fecal incontinences need further detailed study with large sample size.

## Competing interests

The authors have declared that no conflicts of interest with respect to the authorship and/or publication of this research paper.

## Authors’ contributions

YW: Participated in the design of the study, data collection, analysis and interpretations of the findings, drafting the manuscript and write up. DM: participated in the conception and design of the study, analysis and interpretation of the findings. AA: Participated in the conception and design of the study, data analysis and interpretations of the findings. AA: Participated in the conception and design of the study, data analysis and interpretations of the findings. GY: Participated in the conception and design of the study, data analysis and interpretations of the findings. TG: Participated in the conception and design of the study and data collection. AA: Participated in the conception and design of the study, data analysis and interpretations of the findings. DD: Participated in the conception and design of the study, data analysis and interpretations of the findings. GF: Participated in the conception and design of the study, data analysis and interpretations of the findings. All authors reviewed and approved the final manuscript.

## Pre-publication history

The pre-publication history for this paper can be accessed here:

http://www.biomedcentral.com/1472-6874/14/12/prepub
